# Editorial Note

**Published:** 1946

**Authors:** 


### Editorial Note

**University of Bristol £300,000 Extension Scheme.***

In honour of Mr. Winston Churchill, Chancellor of the University of Bristol for nearly twenty years, an appeal for £300,000 has been launched to cover the cost of a great university centre on St. Michael's Hill, closely adjacent to the present University buildings, and the halls of residence focussed around Wills Hall, Stoke Bishop.

The appeal was launched at a meeting on October 30th in the great hall of the University, at which the Lord Mayor Alderman James Owen presided, supported by the Sheriff Mr. Harry Crook, the Vice-Chancellor Sir Philip Morris, members of the Court, Council, Senate and Alumni ; Lord Dulverton, Lord Bledisloe, Admiral Sir James Somerville, and many leading West Country men. It was announced that before the appeal had been launched subscriptions totalling £136,432 had been promised, including £50,000 from Lord Dulverton and £25,000 from the Imperial Tobacco Company, of which he is Chairman.

The guest of honour was Sir Henry Tizard, President of Magdalen College Oxford. He said it was now generally expected that the Universities of England and Wales should double the number of their students within ten years if possible. The view had been expressed that doubling would mean lowering standards, but he was convinced there was no need for that if secondary education developed as they all hoped it would. It was nonsense, he said, to suggest that there would be no occupations for graduates.

The general opinion was that the extension of the University was desirable and practicable. " Bristol has a clear duty and a great opportunity," he continued. " It seems to me it has all the assets except one—finance—to build up a University which can in time rival the ancient cities of learning at Oxford and Cambridge,

* *Reprinted from* "The Times," Wednesday, October 30th, 1946.

not only to the benefit of the country, but even to the benefit of Oxford and Cambridge.

" Bristol is worthy of a residential university of first rank. My rough estimate of the cost of creating a great University for Bristol, of 3,000 or more students, of whom every single one would spend at least one or two years in university residence, would be at least £5,000,000. I am asked if this policy is likely to commend itself to the Government. Why should not the Government in these Socialist times find the money ? It is logical, but in practice much is to be said against it. The need to preserve the essential autonomy of the Universities, we are convinced, is vital. We should show we are prepared to find a large proportion ourselves."

The appeal is based largely on the pressing need for residential accommodation for students, who by October of next year are expected to number 3000 : and it is proposed to provide additional halls of residence named after Mr. Churchill, for both men and women. New Medical and Engineering schools and a school of Veterinary Science are also planned.

